# Profiling the interactome of protein kinase C ζ by proteomics and bioinformatics

**DOI:** 10.1186/s12953-018-0134-8

**Published:** 2018-02-26

**Authors:** Chunyu Hou, Yuan Li, Huiqin Liu, Mengjiao Dang, Guoxuan Qin, Ning Zhang, Ruibing Chen

**Affiliations:** 1 Tianjin Medical University Cancer Institute and Hospital, National Clinical Research Center for Cancer, Key Laboratory of Cancer Prevention and Therapy, Tianjin’s Clinical Research Center for Cancer, Tianjin, 300070 China; 2 Department of Genetics, School of Basic Medical Sciences, Tianjin Medical University, Tianjin, 300070 China; 3 School of Microelectronics, Tianjin University, Tianjin, 300072 China

**Keywords:** Cancer, PKCζ; LC-MS/MS, Proteomics, Bioinformatics, Protein-protein interaction

## Abstract

**Background:**

Protein kinase C ζ (PKCζ), an isoform of the atypical protein kinase C, is a pivotal regulator in cancer. However, the molecular and cellular mechanisms whereby PKCζ regulates tumorigenesis and metastasis are still not fully understood. In this study, proteomics and bioinformatics analyses were performed to establish a protein-protein interaction (PPI) network associated with PKCζ, laying a stepping stone to further understand the diverse biological roles of PKCζ.

**Methods:**

Protein complexes associated with PKCζ were purified by co-immunoprecipitation from breast cancer cell MDA-MB-231 and identified by LC-MS/MS. Two biological replicates and two technical replicates were analyzed. The observed proteins were filtered using the CRAPome database to eliminate the potential false positives. The proteomics identification results were combined with PPI database search to construct the interactome network. Gene ontology (GO) and pathway analysis were performed by PANTHER database and DAVID. Next, the interaction between PKCζ and protein phosphatase 2 catalytic subunit alpha (PPP2CA) was validated by co-immunoprecipitation, Western blotting and immunofluorescence. Furthermore, the TCGA database and the COSMIC database were used to analyze the expressions of these two proteins in clinical samples.

**Results:**

The PKCζ centered PPI network containing 178 nodes and 1225 connections was built. Network analysis showed that the identified proteins were significantly associated with several key signaling pathways regulating cancer related cellular processes.

**Conclusions:**

Through combining the proteomics and bioinformatics analyses, a PKCζ centered PPI network was constructed, providing a more complete picture regarding the biological roles of PKCζ in both cancer regulation and other aspects of cellular biology.

**Electronic supplementary material:**

The online version of this article (10.1186/s12953-018-0134-8) contains supplementary material, which is available to authorized users.

## Background

Protein kinase C (PKC) is a family of protein serine/threonine kinases that are involved in the regulation of diverse biological processes, including gene expression, cell differentiation, apoptosis, proliferation, cytoskeleton organization, cell migration, etc. [1–3]. Based on their distinct features, PKCs can be divided into three subtypes [4]: the conventional or classic PKCs, such as PKC α, β, γ; the novel PKCs, such as PKCδ, ε, η, θ, and the atypical PKCs, including PKCζ and PKCλ. All the three subtypes of PKCs have conserved C terminus kinase domain, but the N terminus regulatory domain varies greatly among different subtypes. For example, the atypical PKCs lack the calcium binding domain and one-half of the C1 homologous domain, therefore do not need diacylglycerol or calcium for their activation, but are dependent on lipid components, such as phosphatidylinositols (PIs) [5, 6].

The atypical PKCs, particularly the ζ isoform, have been implicated in numerous essential signaling events regulating tumorigenesis and cancer progression [7]. For example, earlier studies have shown that PKCζ could bind with different protein regulators and adaptors to regulate the NF-κB pathway and control cell apoptosis [8–10]. PKCζ is also reported to translocate to the nucleus under genotoxic conditions, where it regulates cell viability through the suppression of the apoptosis pathway and induces chemoresistance [11, 12]. In addition, accumulating evidence have shown that PKCζ plays a pivotal role in the regulation of cancer cell migration and invasion [13]. During breast cancer metastasis, EGF induces tumor cells intravasation from primary sites into circulation and SDF-1 mediates their extravasation to the secondary sites [14, 15]. PKCζ plays a regulatory role in both EGF and SDF-1 induced chemotaxis signaling pathways [16, 17]. In glioblastoma cells, pancreatic cancer cells and lung cancer cells, knockdown of PKCζ dramatically reduces cell migration and invasion through regulation of the activities of numerous signaling molecules related with cytoskeleton rearrangement and cell adhesion, including cofilin, LIN-11, Isl1 and MEC-3 protein domain kinase (LIMK) and β-integrin [18, 19]. However, the roles of PKCζ in neoplasia appear to be controversial. A number of clinical studies have shown that the expression levels of PKCζ vary among different types of tumor [7]. PKCζ can inhibit Ras-induced tumorigenesis, and such tumor suppression effect is severely inhibited by the PKCζ S514F mutation [20]. PKCζ is also reported to phosphorylate c-Myc and suppress its activity, therefore inhibit tumorigenesis [21]. Furthermore, a recent study has implicated PKCζ in the regulation of tumor metabolism. PKCζ deficiency promotes the reprogramming of tumor metabolism to utilize glutamine through the serine biosynthetic pathway in the absence of glucose [22]. These evidence have shown that PKCζ is involved in majority of the cancer hallmarks and modulate tumorigenesis through coordinating diverse molecular pathways. However, whether PKCζ is a pro- or anti-neoplastic protein is still under debate, and further investigation is required to clarify its roles in different tumors.

The molecular mechanism by which PKCζ participates in the regulation of cancer biology is largely dependent on its dynamic interactions with other proteins. For example, it has been reported that PKCζ can modulate the NF-κB signaling pathway through interaction with p62 and par-4 [10, 23, 24]. Our previous study shows that rictor, a component of the mTOR complex 2, can bind with PKCζ and mediates PKCζ dependent breast cancer metastasis [25]. Therefore, to establish the interactome of PKCζ is crucial to understand the molecular mechanism whereby PKCζ regulates these signaling events.

In this study, proteomics and bioinformatics analyses were combined to establish a protein-protein interaction (PPI) network associated with PKCζ. Proteins complexes associated with PKCζ from human breast cancer cell line MDA-MB-231 were purified with co-immunoprecipitation and analyzed by LC-MS/MS for protein identification. Two biological replicates and two technical replicates were analyzed. The observed proteins were filtered using the CRAPome database to eliminate the potential false positive identifications. For bioinformatics analysis, PKCζ was searched against the STRING PPI database. The proteomics identification and database search results were combined for network construction. A PKCζ centered PPI network was constructed, providing a more complete picture regarding the biological roles of PKCζ in the regulation of cancer hallmarks. Furthermore, molecular and cellular biology assays, such as immunofluorescence, co-immunoprecipitation (Co-IP), Western blotting, and cell migration assay were performed to study the biological implications of the interaction between PKCζ and protein phosphatase 2 catalytic subunit alpha (PPP2CA).

## Methods

### Antibodies and reagents

Mouse monoclonal antibody against Flag, anti-Flag antibody conjugated agarose beads, dithiothreitol (DTT), iodoacetamide (IAA) were from Sigma-Aldrich (St. Louis, MO, USA). Mouse monoclonal antibody against β-actin was from Santa Cruz (Santa Cruz, CA, USA). Mouse monoclonal antibody against PKCζ and rabbit polyclonal antibody against PPP2CA were from Cell Signaling Technology (Danvers, MA, USA). Lipofectamine 2000, BCA reagents, and Protein G agarose beads were purchased from Invitrogen. Enhanced chemiluminescence reagents were from Pierce Biotechnology. Protease Inhibitor Cocktail tablets were from Roche Diagnostics (Indianapolis, IN, USA). Sequencing grade modified trypsin was purchased from Promega (Madison, WI, USA). LC-MS grade water and acetonitrile were bought from Merck (White-house Station, NJ, USA).

### Clinical sample analysis

The expression data of PKCζ and PPP2CA in breast cancer was obtained through the cBio Cancer Genomics Portal (http://cbioportal.org), an open platform for exploring multidimensional cancer genomics data (TCGA) [26]. For survival curve analysis, the median mRNA expression level of PKCζ was used as a cut-off value to divide the data into two groups. The survival curves of the high-expression and low-expression groups were compared using the log-rank tests.

### Cell culture, plasmid and transfection

Human breast cancer cell line MDA-MB-231 and MCF-7 were obtained from American Type Culture Collection. Cells were cultured in DMEM supplemented with 10% fetal bovine serum and 1% glutamine Pen-Strep solution at 37 °C and 5% CO_2_.

Flag-PKCζ was amplified by PCR and cloned into vector pcDNA 3.1. The Flag-PKCζ plasmids were then transfected into MDA-MB-231 using Lipofectamine 2000 and Flag-PKCζ stable cell line was established. The expression of Flag-PKCζ fusion protein was confirmed by Western blotting with both anti-PKCζ and anti-Flag antibodies.

For PPP2CA knockdown, three human PPP2CA-siRNA duplexes were designed and synthesized by RiboBio (Guangzhou, China). Non-targeting siRNA was also synthesized by RiboBio and used as negative control. The siRNAs was transfected into the cells by using X-tremeGENE siRNA Transfection Reagent (Roche, Indianapolis, IN, USA).

### Coimmunoprecipitation (Co-IP)

Co-IP was performed to purify PKCζ and its interacting proteins. Briefly, cells were cultured to 80%–90% confluence and starved with serum free medium for 12 h. Cellular proteins were extracted with lysis buffer (40 mM Tris, 120 mM NaCl, 1% Triton X-100, 1 mM NaF, 1 mM Na_3_VO_4_) supplemented with protease inhibitor cocktail. Total protein concentration of the extract was measured with BCA assay. The cell extracts were precleared with protein G agarose beads, and then PKCζ and its interacting proteins were isolated with anti-Flag antibody conjugated agarose beads, followed by Western blotting or mass spectrometric analysis.

### SDS-PAGE and western blotting

Proteins were eluted from the agarose beads by incubation with the SDS-PAGE loading buffer in boiling water bath for 10 min. For Western blotting, proteins separated by SDS-PAGE were transferred onto polyvinylidene fluoride membranes using a wet electro-blotter. The membranes were incubated with primary antibodies at 4 °C overnight, and followed by incubation with secondary antibodies at room temperature for 1 h. Bound antibodies were detected by the ECL immumoblotting detection reagent.

### Proteolysis and mass spectrometric analysis

PKCζ interacting proteins were eluted from agarose beads with 6 M urea in 25 mM ammonium bicarbonate buffer, pH 8. The samples were reduced by incubating with 10 mM DTT at 37 °C for 1 h. The reduced proteins were alkylated for 1 h in darkness with 40 mM iodoacetamide. The alkylation reaction was quenched by adding DTT to a final concentration of 50 mM. The urea in the solution was exchanged to 25 mM ammonium bicarbonate buffer by centrifugation using 3 kDa ultrafiltration devices (Millipore). Next, trypsin was added at a 50:1 protein to trypsin mass ratio, and the samples were incubated at 37 °C overnight for the digestion to complete.

A nanoelectrospray ionization (nESI) LTQ XL linear ion trap mass spectrometer (Thermo Electron Corp) coupled with nanoLC system was used for protein identification. Two biological replicates and two technical replicates were analyzed. The LTQ mass spectrometer was operated in a data-dependent mode in which an initial MS scan recorded the mass range of m/z 400–2000, and the ten most abundant ions were automatically selected for CAD fragmentation. The spray voltage was set as 2.5 kV. The normalized collision energy was set at 35% for MS/MS. Raw LTQ data was searched against the IPI human protein database using SEQUEST algorithm embedded in the Protein Discoverer 1.3 Software (Thermo Electron Corp). The following parameters were applied during the database search: 1 Da precursor mass error tolerance, 1 Da fragment mass error tolerance, static modifications of carbamido methylation for all cysteine residues and oxidation modifications of methionine residues. One missed cleavage site of trypsin was allowed. A reversed database was searched to evaluate the level of false discovery rate (FDR). FDR < 0.05 was used as filtering criteria for proteins with multiple tryptic peptides, and FDR < 0.01 was used for proteins identified with single tryptic peptide. Proteins with shared tryptic peptides were grouped and treated as one.

### Bioinformatics analysis

The CRAPome database is a web-accessible (http://www.crapome.org/) repository of negative-control AP-MS experiments. To eliminate potential false positive identifications, proteins identified using LC-MS/MS were uploaded to the CRAPome database and score ≥ 20 was set as threshold for false positives. Known PPI information was obtained from both literature and several public PPI databases, including STRING (http://string90.embl.de/), Biogrid (http://thebiogrid.org/), MINT (http://mint.bio.uniroma2.it/), IntAct (http://www.ebi.ac.uk/intact/) and HPRD (http://www.hprd.org/). The PPI data obtained through database search and mass spectrometric analysis was then integrated in Excel and imported into Cytoscape v2.8.3 (http:// www.cytoscape.org/) for network visualization. Gene ontology annotation was conducted by the PANTHER database program (http://www.pantherdb.org/) [27]. Pathway analysis was performed using DAVID (https://david.ncifcrf.gov/).

The interactome network analysis was conducted using the Systems Biology and Evolution MATLAB Toolbox (SBEToolbox) and Cytoscape. Several characteristic properties of the constructed network were computed, including node-specific degree k, clustering coefficient, and small-world index. The power-law degree distributions and adjacency matrices of the networks were generated using MATLAB. The rich-club coefficient is computed as described by others [28, 29]. Briefly, we generated 1000 comparable random networks with equal size and the same degree distribution by rewiring to calculate the normalized rich-club coefficient. When the normalized rich-club coefficient is greater than 1, it indicates the rich-club organization in the network is significant.

### Immunofluorescence

Cells were cultured on six-well chamber slides. At the time of harvest, cells were fixed with 4% paraformaldehyde and then permeabilized with 0.01% Triton X-100 for 10 min. The cells were incubated with primary antibodies at 4 °C overnight, followed by staining with Alexa Fluor 488 and 546–conjugated secondary antibodies for 1 h at room temperature. All samples were treated with 4′,6-diamidino-2-phenylindole (DAPI) dye for nuclear staining. A Nikon C2 Plus confocal microscope was applied for imaging.

### Quantitative reverse transcription PCR (qRT-PCR)

Total RNA was isolated by Trizol reagent (Life Technologies, Carlsbad, CA, USA). Reverse transcription reactions were performed with 5 μg of total RNA using a FastQuant RT kit (TIANGEN, Beijing, China) according to the manufacturer’s protocols. The cDNA was subjected to quantitative real-time PCR (qRT-PCR) using the SYBR Green PCR Kit (TIANGEN, China) and the assay was performed on an ABI PRISM 7500 Sequence Detector. Expression data were uniformly normalized to GAPDH as an internal control, and the relative expression levels were evaluated using the 2^-ΔΔCt^ method. The primer sequences for PPP2CA were 5′-GAT CTT CTG TCT ACA TGG TGG TCT C-3′ (Forward) and 5’-ACA CAT TGG ACC CTC ATG GGG AA-3′ (Reverse). GAPDH was used as an internal control (forward: 5’-TGC ACC ACC AAC TGC TTA GC-3′; reverse: 5’-GGC ATG GAC TGT GGT CAT GAG-3′).

### Cell migration assay

In wound healing assay, MDA-MD-231 cells were seeded in 6-well plates and grown until 80–90% confluence. The cells were scratched with a pipette tip in the middle of the plate, washed with PBS to remove the detached cells and incubated in a medium containing 1% FBS. The wound closure was monitored microscopically at different time-points and photographed at 0 and 24 h respectively.

### Statistical analysis

SPSS version 17.0 software were performed for statistical analyses and Prism version 5.0 (GraphPad) were used to plot to show mean and standard deviation (SD). Student’s *t* test was performed for comparison. All statistical tests were two-sided and *P* values were considered statistically significant for *P* ≤ 0.05.

## Results

**Fig. 1:**
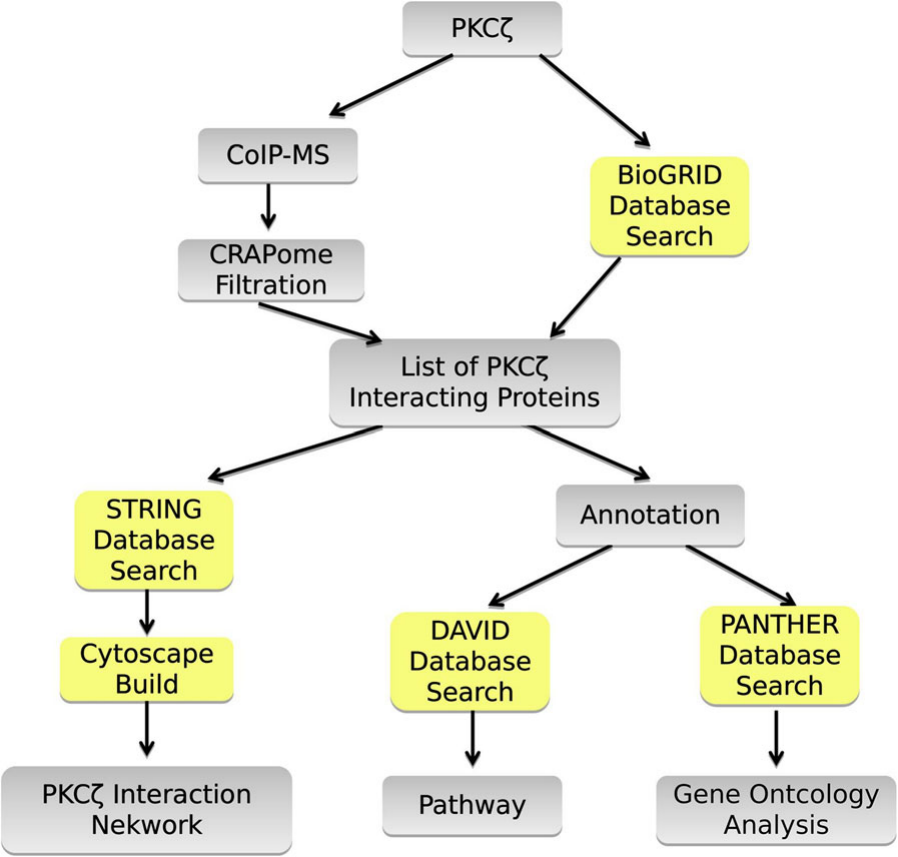
The overview of the experimental workflow. PKCζ interacting proteins characterized using CoIP-MS detection. The observed proteins were filtered using the CRAPome database to eliminate the potential false positives. The proteins identified using mass spectrometry were combined with PKCζ interacting proteins identified through literature mining and PPI database search. The complete list of PKCζ interacting proteins were analyzed using the PANTHER database. Furthermore, the interaction between each protein was obtained using STRING PPI database search, and the results were uploaded into Cytoscape for network construction

PKCζ has previously been implicated in different hallmarks of cancer [7]. We first analyzed the expression of PKCζ in breast cancer by searching the TCGA database. Survival analysis based on RNA sequencing data from 1445 patients in TCGA database showed that the over-expression of PKCζ is associated with poor prognosis (*P* = 0.0011, Additional file 1: Figure S1A). To better understand the biological roles of PKCζ in the diverse signaling pathways regulating cancer, proteomics and bioinformatics analyses were combined to establish a PPI network associated with PKCζ. As shown in Fig. 1, PKCζ interacting proteins were isolated with Co-IP and analyzed by LC-MS/MS for protein identification. The observed proteins were filtered using the CRAPome database to eliminate the potential false positives. The MS identification results were combined with PPI database search to construct the PKCζ interactome network. The PKCζ interacting proteins were further analyzed using the PANTHER database and the DAVID database.

### Characterization of PKCζ interacting proteins

**Fig. 2:**
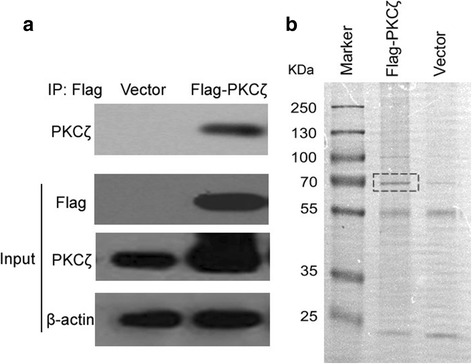
Isolation of PKCζ interacting proteins. **a** Western blotting analysis of PKCζ immunoprecipitates. MBA-MD-231 cells were transfected with vector control and Flag-PKCζ plasmids, and stable clones were cultured. The presence of Flag and PKCζ in these cells were detected by Western blotting. Co-IP was performed using anti-Flag conjugated beads. **b** SDS-PAGE separation of the Flag-PKCζ immunoprecipitates. The gel was visualized using silver staining

Firstly, CoIP-MS was employed to identify PKCζ interacting proteins. MDA-MD-231 breast cancer cells were transfected with Flag-PKCζ fusion protein and stable clones were cultured. As shown in Fig. 2a, Western blotting showed that the stable clone cells successfully expressed Flag-PKCζ. PKCζ interacting proteins were isolated using anti-Flag antibody from the Flag-PKCζ cells. PKCζ was enriched in the immunoprecipitates as detected with Western blotting and SDS-PAGE (Fig. 2). Using LC-MS/MS analysis, 233 proteins were detected in the Flag immunoprecipitates. After CRAPome filtration, 106 proteins were identified as potential PKCζ interacting proteins (Additional file 1: Table S1). Some of these proteins are known PKCζ interacting proteins, such as sequestosome 1/p62 (SQSTM1) and complement component C1qbinding protein (C1QBP). PPI Databases search was also performed to achieve comprehensive identification of PKCζ interacting proteins. Combining literature mining and searching through several PPI databases, including BioGrid, InACT, STRING, MINT and HPRD, we were able to obtain 77 PKCζ interacting proteins (Additional file 1: Table S2). This set of data included some of the well known PKCζ interacting proteins, such as AKT and several other isoforms of PKCs.

### Gene ontology analysis of the PKCζ interacting proteins

**Fig. 3:**
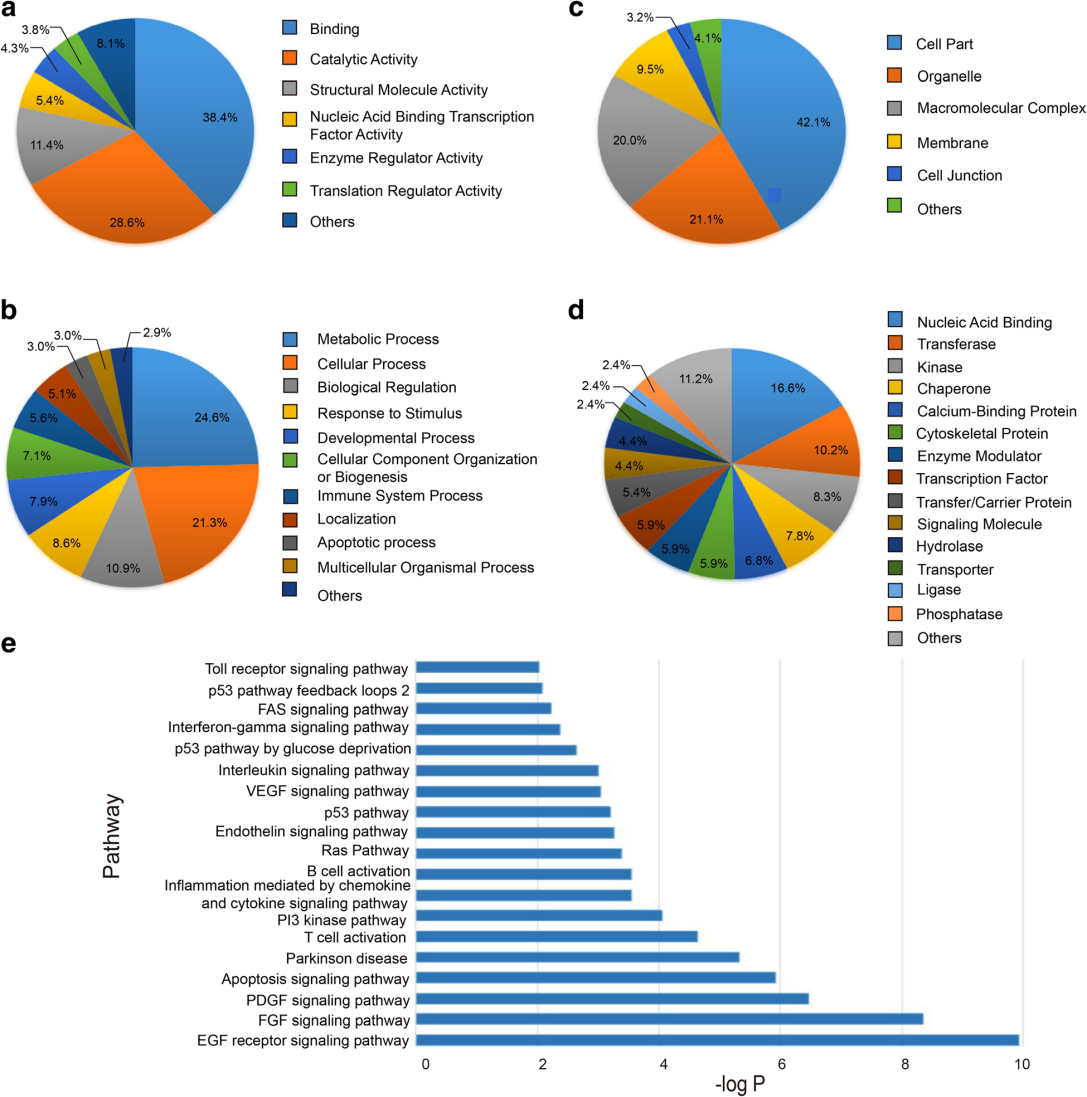
Diagram showing the assignment of gene ontology terms to the identified PKCζ interacting proteins: (**a**) molecular functions. **b** biological process. **c** cellular component. **d** protein classes. Analysis was performed using the PANTHER database program (www.pantherdb.org). **e** The signaling pathways associated with the PKCζ interacting proteins

The combined PKCζ interacting proteins were further analyzed for gene ontology annotation. As shown in Fig. 3a, molecular function analysis revealed that most of the identified proteins were related with protein binding (38.4%), catalytic activity (28.6%), structural molecule activity (11.4%), nucleic acid binding transcription regulation activity (5.4%), and enzyme regulator activity (4.3%). As shown in Fig. 3b, biological process analysis showed that PKCζ interacting proteins are associated with metabolic process (24.6%), cellular process (21.3%), biological regulation (10.9%), response to stimulus (8.6%), developmental process (7.9%), cellular component organization or biogenesis (7.1%), and immune system process (5.6%). Cellular component analysis showed that these non-specific binding proteins were from various regions of the cell, such as cell part (42.1%), organelle (21.1%) and macromolecular complex (20.0%), and membrane (9.5%) (Fig. 3c). As shown in Fig. 3d, the major protein classes included nucleic acid binding (16.6%), transferase (10.2%), kinase (8.3%), chaperone (7.8%), calcium binding protein (6.8%), and cytoskeletal proteins (5.9%).

Associated signaling pathways were analyzed using DAVID, and the related signaling pathways with *P* value ≤0.01 were shown in Fig. 3e and Additional file 1: Table S3. The top related signaling pathway (with 19 associated proteins) is the EGF signaling pathway. The FGF and PDGF signaling pathways are also relevant, and many proteins in these two pathways overlap with the EGF pathway. The next significantly related signaling pathway is the apoptosis pathway. PKCζ interacts with 14 proteins from the apoptosis pathway, such as the inhibitor of nuclear factor kappa-B kinase subunit beta (IKBKB). The inflammation mediated by chemokine and cytokine signaling pathway is also highly relevant. The pathway analysis results are highly consistent with the known functions of PKCζ.

### Construction and analysis of the PKCζ interactome network

**Fig. 4:**
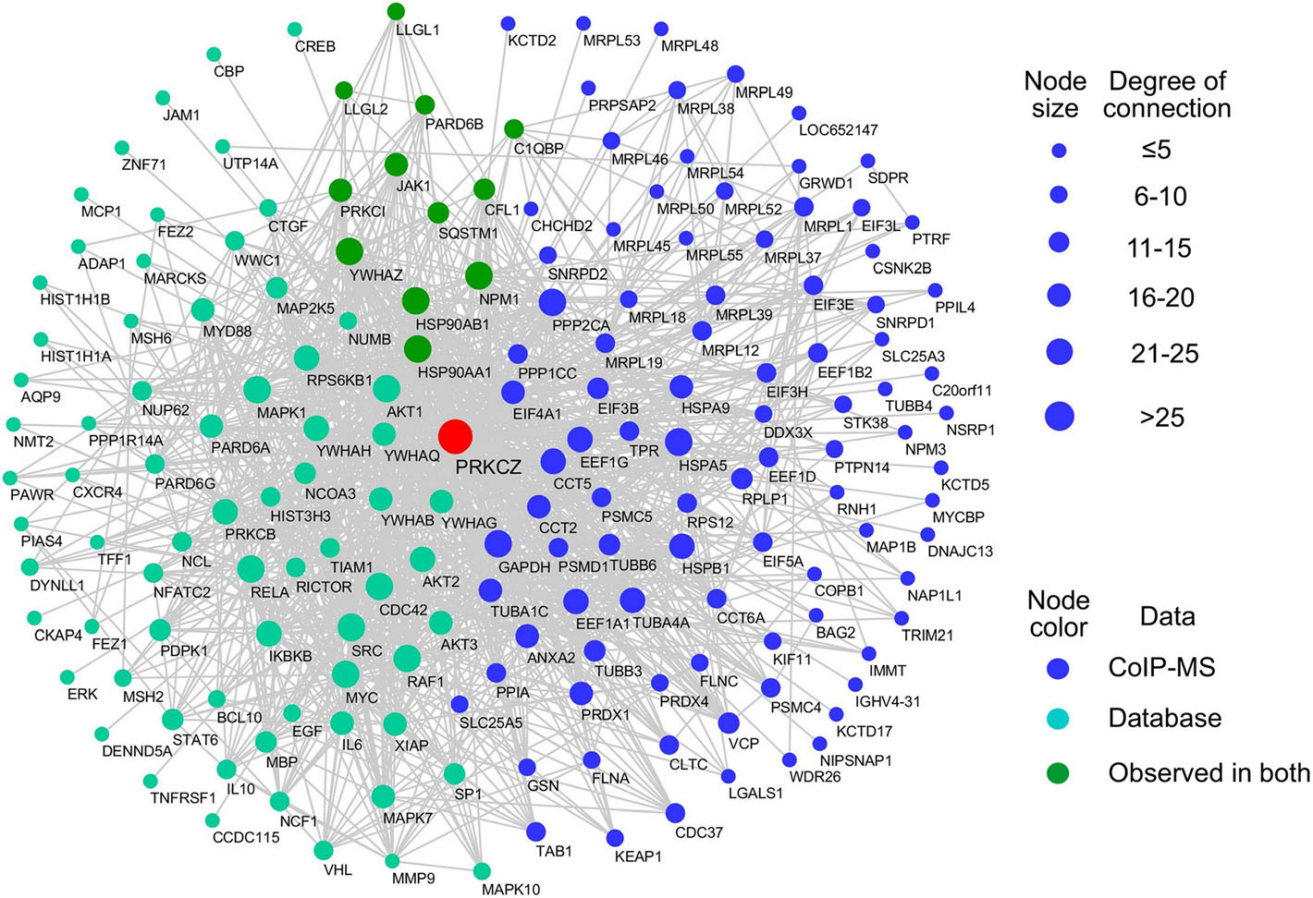
PPI network of the PKCζ interacting proteins. PKCζ interacting proteins (from Additional file 1: Table S1) and their interactions are shown as nodes and edges. Proteins identified using mass spectrometry and database search were shown in different colors as indicated in the graph. Node size reflects the interaction degree as indicated. The network was constructed using Cytoscape

The PKCζ interacting proteins obtained through mass spectrometry analysis and database mining were searched against the STRING database for interaction information and imported to Cytoscape for network construction. As shown in Fig. 4, a highly connected network composed of 183 proteins and 1225 connections was mapped. About half of the mapped proteins were from data search and literature, and half of the proteins were identified with mass spectrometry analysis. Only 12 proteins were observed using both methods, including C1QBP, SQSTM1, JAK1, LLGL1, etc.

Most of the known PKCζ interacting proteins from literature and databases were identified using Western blotting or yeast two hybrid. These methods may scope different types of proteins compared to mass spectrometric analysis.

**Fig. 5:**
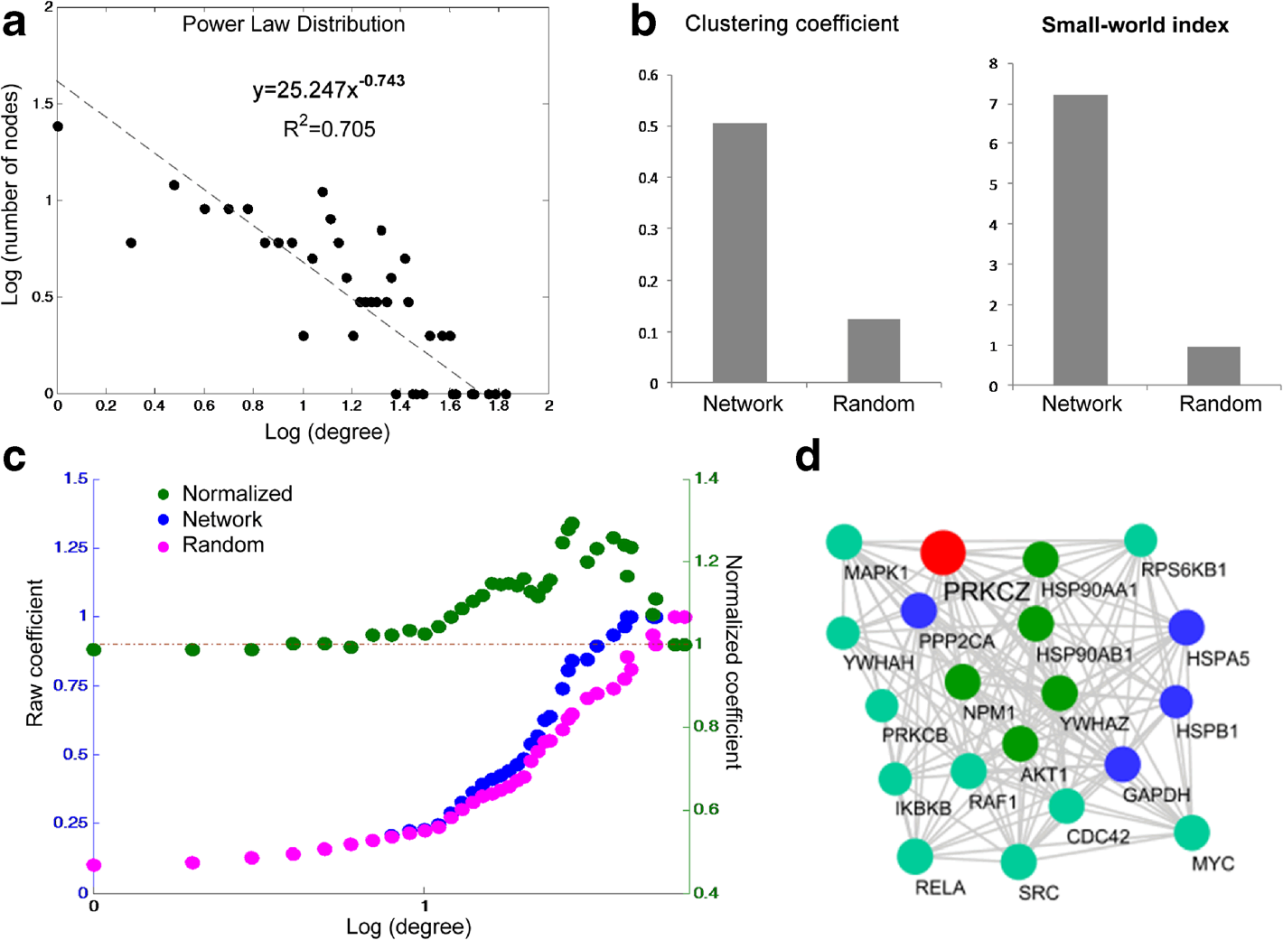
Properties of the PKCζ PPI network. **a** Power-law distribution curve of the PKCζ network shows a negative correlation between the number of nodes (y axis) and the node-specific degree (x axis), Pearson correlation coefficient ρ = − 0.856. **b** Identification of the small-world organization within the PKCζ PPI network. Clustering coefficient of constructed network was significantly higher as compared with that of the random networks. The small-world coefficient was 7.2. **c** Raw rich-club coefficient of the constructed PKCζ PPI network (blue) and random network (red) plotted against the left vertical axis. Normalized rich-club coefficient for the network (green) plotted against the right vertical axis. **d** The sub-network of the rich-club organization within the PKCζ network. Proteins are indicated with circles with different colors as used in Fig. 5, and the interactions between proteins are indicated with grey edges

As shown in Fig. 4, some of these proteins are highly connected in the constructed network (degree of connection was indicated with node size). To further understand the properties of the constructed PKCζ interactome network, a rich-club analysis was conducted. A rich club is a set of high-degree nodes that are more densely interconnected than predicted by the node degrees alone [29]. The rich-club nodes may form a hub that is used by the other components in the network to influence each other. As shown in Fig. 5a, the PKCζ interactome network exhibits a power-law degree distribution consistent with being a scale-free network. The results suggest that the frequency of nodes negatively correlates with the connection degree indicating a few number of nodes have the majority of the interactions in the network and therefore may form a connection hub. Further analysis of the network clustering coefficient showed that the PKCζ interactome network has relatively higher clustering coefficient and higher small-world index as compared to the random networks (Fig. 5b). In addition, the presence of a rich-club organization within the PKCζ interactome network was characterized. To investigate the significance of the discovered rich-club, the rich-club coefficient of the PKCζ interactome network was compared to that of 1000 randomly generated networks with similar degree distribution. The normalized rich-club coefficient reveals the presence of a significant rich-club between degrees 11 and 175 and a peak at degree 29 (Fig. 5c). The sub-network of nodes with degrees corresponding to the highest normalized rich-club coefficient (above 1.2) was shown in Fig. 5d. This core network contained 20 nodes and 141 edges. The rich-club network includes some of the most well known PKCζ interacting proteins, such as AKT1, IKBKB, MAPK1, etc. These proteins may play a more influential role within the overall interactome network of PKCζ.

### The interaction between PPP2CA and PKCζ

**Fig. 6:**
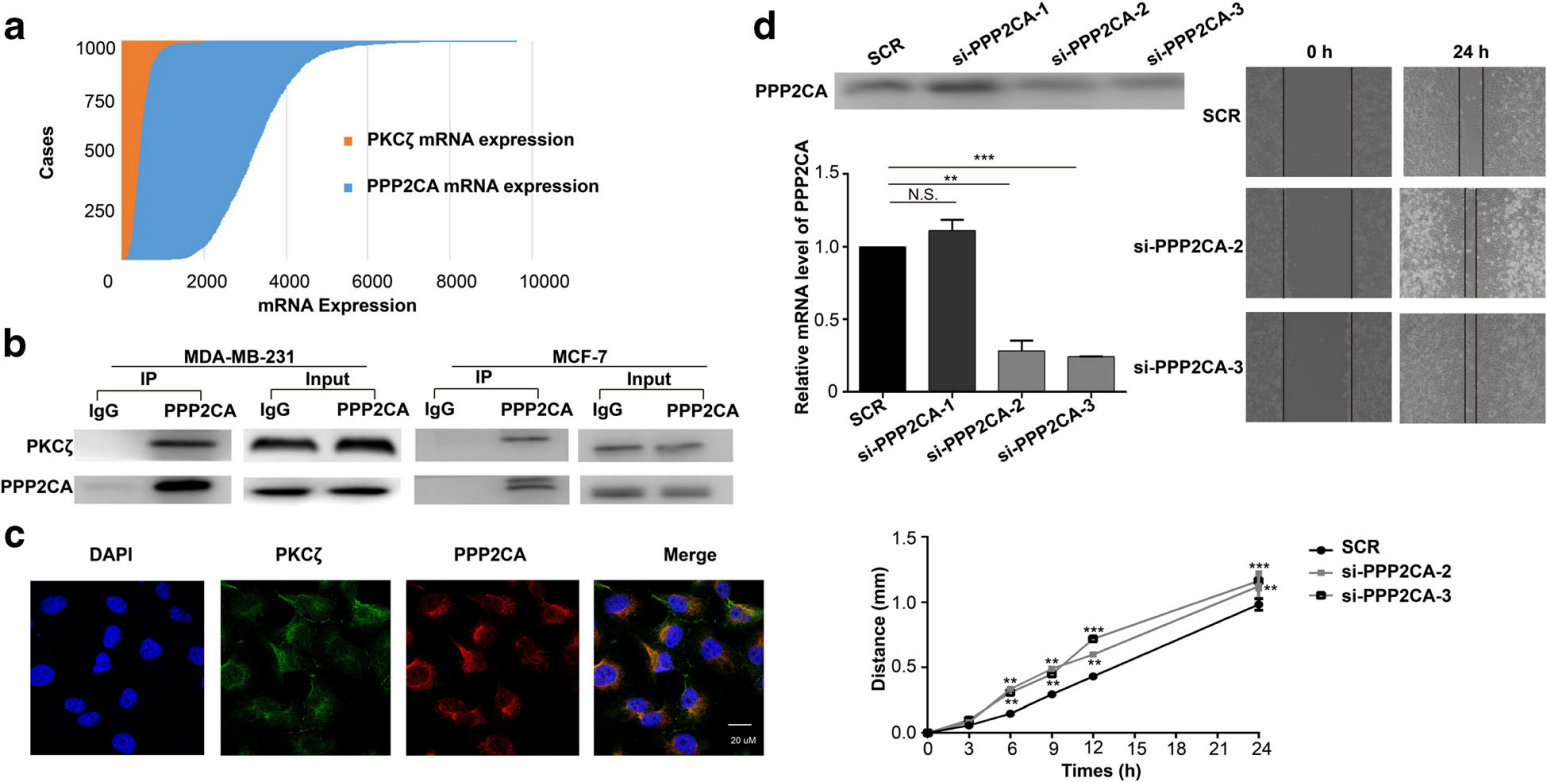
The interaction between PKCζ and PPP2CA. **a** The mRNA levels of PPP2CA and PKCζ in breast cancer samples from the TCGA database. **b** Western blotting analysis of the PPP2CA immunoprecipitates from MDA-MB-231 and MCF-7 cells. Co-IP was performed using anti-PPP2CA antibody conjugated protein G agarose beads. Normal IgG was used as control. **c** Immunofluorescence analysis of PKCζ and PPP2CA in MDA-MB-231 cells. Cell nuclei were stained with DAPI. **d** PPP2CA knockdown in MDA-MB-231 cell enhanced cell migration. The efficiency of PPP2CA knockdown was examined by qRT-PCR and Western blotting. Bar; mean; error bar: SD (*P < 0.05, **P < 0.01, ***P < 0.0001, by student’s *t*-test)

PPP2CA is one of the four newly identified PKCζ interacting proteins that present in the rich-club core network. PPP2CA is a component of the protein phosphatase 2A (PP2A), an important and ubiquitously expressed serine threonine phosphatase that regulates many cellular processes by dephosphorylating critical cellular molecules like AKT, P53, c-Myc and β-catenin [30, 31]. The PP2A hetero-trimer consists of a catalytic core comprised of the catalytic A and C subunits as well as a regulatory B subunit that controls substrate specificity and cellular localization [32]. The interaction between PPP2CA and PKCζ may be very important for their functions in different biological processes. Therefore, we sought to validate their interaction. To investigate the correlation of these two studied proteins, we analyzed their expressions in 1145 breast cancer samples from TCGA. As shown in Fig. 6a, PPP2CA and PKCζ were both up-regulated in breast cancer tumor tissues. Co-IP and Western blotting showed that PPP2CA could indeed bind to PKCζ in two types of human breast cancer cell line, including MDA-MB-231 and MCF-7 (Fig. 6b). In addition, immunefluorescence showed that these two proteins both localized in the cytoplasm (Fig. 6c). Our previous study has shown that PKCζ is a key regulatory molecule that promotes cell migration and breast cancer metastasis [16]. To understand the biological impact of the interaction between PKCζ and PPP2CA, we knocked down PPP2CA from MDA-MB-231 cells and investigated its effect on cell migration. Three siRNAs were used to silence PPP2CA from MDA-MB-231 cells. qRT-PCR and Western blotting showed that sequence #2 and #3 could efficiently knocked down the levels of PPP2CA from the cells. Results from cell migration assay showed that down-regulation of PPP2CA increased cell migration in MDA-MB-231 cells (Fig. 6d). Taken together, the results implicated that PPP2CA may affect breast cancer cell migration through interacting with PKCζ.

## Discussion

In this study, we combined proteomics and bioinformatics analysis to construct a comprehensive PKCζ interactome network consisting of 178 proteins and 1225 connections. This map is important for further understanding the complicated roles PKCζ plays in the diverse biological processes regulating cancer. Previous studies have suggested that the activation of PKCζ is controlled by phosphoinositide 3-kinase (PI3K) and PDK1 [33, 34], which are major downstream effectors of receptor tyrosine kinases, including EGFR, PDGFR, FGFR, VEGFR, etc. Consistent with these reports, our results showed that the top 3 related signaling pathways are EGF, FGF and PDGF pathways. It is well known that activated receptor tyrosine kinases regulate cellular processes through two major pathways: PI3K/Akt and Ras/MAPK signaling. In this study, we found that several PKCζ binding proteins are associated with these two cellular signaling pathways. In addition, the results showed that multiple PKCζ binding proteins are associated with chemokine and cytokine signaling pathways. In agreement with these observations, previous studies have shown that PKCζ is involved in the regulation of directional cell migration, such as chemotaxis, which plays a critical role in cancer cell invasion and metastasis [35–37]. Intensive studies indicate that PKCζ is a key mediator of EGF-induced chemotaxis and is required for cancer cell metastasis [1, 38–40]. Together, this study provides a detailed map of the PKCζ centered PPIs and their coordination that regulate these pathways.

The core network achieved through rich-club analysis indicated that 20 proteins are highly connected with PKCζ, such as AKT, MAPK1, IKBKB, MYC, etc. These proteins may play a more important role in the PKCζ network. The direct interaction between PKCζ and AKT2 has been implicated in chemotaxis, and AKT2 directly mediates EGF-induced chemotactic signaling pathways through PKCζ [38]. In addition, PKCζ is involved in the MAPK cascade. Through participating in TNF-dependent transactivation of NF-kappa-B through phosphorylating and activating IKBKB kinase, PKCζ leads to degradation of NF-κB inhibitors [6]. Furthermore, decreased phosphorylation of c-Myc at Ser-373 was observed in PKCζ knockout tumors, suggesting PKCζ is a critical regulator of c-Myc [21]. Investigating other proteins mapped in the rich club network and their interactions will be helpful to further elucidate the functions of PKCζ in tumorigenesis and cancer metastasis.

In this study, we validated PPP2CA as a novel PKCζ interacting protein. PPP2CA gene encodes the catalytic subunit C of PP2A, which is one of the four major Ser/Thr phosphatases [41]. PP2A plays critical roles in diverse cellular processes, such as cell proliferation [42], signal transduction [43] and apoptosis [44]. Some of these functions overlap with PKCζ. Intriguingly, the interaction we observed here is between a phosphatase and a kinase, and it has been reported that the activations of both PPP2CA and PKCζ depend on their phosphorylations. Therefore, it is very likely that they could regulate the activities of each other through phosphorylation and de-phosphorylation. It would be interesting to further investigate the biological functions of this interaction and to reveal the underlying molecular mechanism.

## Conclusions

In this study, the PPI network of PKCζ containing 178 nodes and 1225 connections was constructed through combining proteomics and bioinformatics analyses. A comprehensive gene ontology and pathway analysis was performed on the PKCζ interacting proteins. The results suggest that PKCζ may regulate multiple cellular processes through coordinating diverse signaling pathways related with cancer. This study provides a more complete picture regarding the biological roles of PKCζ in both cancer regulation and other aspects of cellular biology.

## Additional file

Additional file 1: Supplemental information. (DOC 78 kb)

## Abbreviations

C1QBP: Complement component C1qbinding protein
Co-IP: Co-immunoprecipitation
DAPI: 4′,6-diamidino-2-phenylindole
DTT: Dithiothreitol
FDR: False discovery rate
GO: Gene ontology
IAA: Iodoacetamide
IKBKB: Inhibitor of nuclear factor kappa-B kinase subunit beta
LIMK: LIM domain kinase
nESI: Nanoelectrospray ionization
PI3K: Phosphoinositide 3-kinase
PIs: Phosphatidylinositols
PKC: Protein kinase C
PKCζ: Protein kinase C ζ
PP2A: Protein phosphatase 2A
PPI: Protein-protein interaction
PPP2CA: Protein phosphatase 2 catalytic subunit alpha
qRT-PCR: Quantitative reverse transcription PCR
SQSTM1: Sequestosome 1/p62
