# Supplementary material for: Profiling the interactome of protein kinase C ζ by proteomics and bioinformatics
Source: Proteome Sci. 2018 Feb 26;16:5. doi: 10.1186/s12953-018-0134-8 (PMC5828088; doi:10.1186/s12953-018-0134-8)
Supplement: Supplementary file 1 — Supplemental information. (DOC 78 kb) [file 12953_2018_134_MOESM1_ESM.doc]

**Table S1**

| **Accession** | **Gene name** | **Description** |
| --- | --- | --- |
| IPI00000643.1 | BAG2 | BAG family molecular chaperone regulator 2 |
| IPI00003362.3 | HSPA5 | 78 kDa glucose-regulated protein |
| IPI00004506.3 | KCTD5 | BTB/POZ domain-containing protein KCTD5 |
| IPI00005705.1 | PPP1CC | Isoform Gamma-1 of Serine/threonine-protein phosphatase PP1-gamma catalytic subunit |
| IPI00005809.7 | SDPR | Serum deprivation-response protein |
| IPI00007188.6 | SLC25A5 | ADP/ATP translocase 2 |
| IPI00007673.4 | CHCHD2 | Coiled-coil-helix-coiled-coil-helix domain-containing protein 2, mitochondrial |
| IPI00008380.1 | PPP2CA | Serine/threonine-protein phosphatase 2A catalytic subunit alpha isoform |
| IPI00008527.3 | RPLP1 | 60S Acidic ribosomal protein P1 |
| IPI00008868.4 | MAP1B | Microtubule-associated protein 1B |
| IPI00010865.1 | CSNK2B | Casein kinase II subunit beta |
| IPI00011937.1 | PRDX4 | Peroxiredoxin-4 |
| IPI00013068.1 | EIF3E | Eukaryotic translation initiation factor 3 subunit E |
| IPI00013122.1 | CDC37 | Hsp90 co-chaperone Cdc37 |
| IPI00013195.1 | MRPL49 | 39S Ribosomal protein L49, mitochondrial |
| IPI00013683.2 | TUBB3 | Tubulin beta-3 chain |
| IPI00013749.3 | PRKCZ | Protein Kinase C zeta type |
| IPI00013917.3 | RPS12 | 40S Ribosomal protein S12 |
| IPI00014230.1 | C1QBP | Complement component 1 Q subcomponent-binding protein, mitochondrial |
| IPI00016634.1 | C20orf11 | Protein C20orf11 |
| IPI00016639.7 | PRKCI | Protein kinase C iota type |
| IPI00018914.2 | PTPN14 | Tyrosine-protein phosphatase non-receptor type 14 |
| IPI00018971.8 | TRIM21 | Isoform 1 of E3 ubiquitin-protein ligase TRIM21 |
| IPI00019025.2 | PARD6B | Partitioning defective 6 homolog beta |
| IPI00021263.3 | YWHAZ | 14-3-3 Protein zeta/delta |
| IPI00022774.3 | VCP | Transitional endoplasmic reticulum ATPase |
| IPI00023161.1 | MRPL46 | 39S Ribosomal protein L46, mitochondrial |
| IPI00023598.2 | TUBB4 | Tubulin beta-4 chain |
| IPI00023860.1 | NAP1L1 | Nucleosome assembly protein 1-like 1 |
| IPI00025512.2 | HSPB1 | Heat shock protein beta-1 |
| IPI00026496.3 | NPM3 | Nucleoplasmin-3 |
| IPI00027096.2 | MRPL19 | 39S Ribosomal protein L19, mitochondrial |
| IPI00027251.1 | STK38 | Serine/threonine-protein kinase 38 |
| IPI00027831.1 | GRWD1 | Glutamate-rich WD repeat-containing protein 1 |
| IPI00061531.4 | MRPL53 | 39SRibosomal protein L53, mitochondrial |
| IPI00064086.5 | EEF1D | Elongation factor 1-delta isoform 4 |
| IPI00106502.5 | KEAP1 | Kelch-like ECH-associated protein 1 |
| IPI00160421.3 | MRPL18 | 39S Ribosomal protein L18, mitochondrial |
| IPI00162330.3 | MRPL37 | 39S Ribosomal protein L37, mitochondrial |
| IPI00178440.3 | EEF1B2 | Elongation factor 1-beta |
| IPI00179473.9 | SQSTM1 | Isoform 1 of Sequestosome-1 |
| IPI00215777.1 | SLC25A3 | Isoform B of Phosphate carrier protein, mitochondrial |
| IPI00216770.1 | PSMC4 | Isoform 2 of 26S protease regulatory subunit 6B |
| IPI00218343.4 | TUBA1C | Tubulin alpha-1C chain |
| IPI00219219.3 | LGALS1 | Galectin-1 |
| IPI00220740.1 | NPM1 | Isoform 2 of Nucleophosmin |
| IPI00295427.5 | MRPL39 | Isoform 1 of 39S ribosomal protein L39, mitochondrial |
| IPI00295851.4 | COPB1 | Coatomer subunit beta |
| IPI00297779.7 | CCT2 | T-complex protein 1 subunit beta |
| IPI00302850.4 | SNRPD1 | Small nuclear ribonucleoprotein Sm D1 |
| IPI00305289.2 | KIF11 | Kinesin-like protein KIF11 |
| IPI00307259.12 | DNAJC13 | DnaJ homolog subfamily C member 13 |
| IPI00329036.2 | MRPL50 | 39S Ribosomal protein L50, mitochondrial |
| IPI00332157.2 | MRPL54 | 39S Ribosomal protein L54, mitochondrial |
| IPI00377224.4 | MRPL52 | 39S Ribosomal protein L52, mitochondrial isoform c |
| IPI00395625.1 | TAB1 | TGF-beta-activated kinase 1 and MAP3K7-binding protein 1 isoform beta |
| IPI00411704.9 | EIF5A | Isoform 1 of Eukaryotic translation initiation factor 5A-1 |
| IPI00413958.5 | FLNC | Isoform 2 of Filamin-C |
| IPI00414676.6 | HSP90AB1 | Heat shock protein HSP 90-beta |
| IPI00440769.3 | KCTD2 | KCTD2 protein (Fragment) |
| IPI00448925.6 | IGHV4-31 | 44 kDa protein |
| IPI00455315.4 | ANXA2 | Isoform 1 of Annexin A2 |
| IPI00455383.4 | CLTC | Isoform 2 of Clathrin heavy chain 1 |
| IPI00456695.1 | PSMD1 | Isoform 2 of 26S proteasome non-ATPase regulatory subunit 1 |
| IPI00472663.2 | MRPL55 | 15 kDa protein |
| IPI00513773.1 | PTRF | Isoform 2 of Polymerase I and transcript release factor |
| IPI00550069.3 | RNH1 | Ribonuclease inhibitor |
| IPI00555749.1 | PSMC5 | Proteasome 26S ATPase subunit 5 variant (Fragment) |
| IPI00640741.1 | PRDX1 | 19 kDa protein |
| IPI00642862.1 | PPIL4 | Peptidyl-prolyl cis-trans isomerase-like 4 |
| IPI00644576.1 | FLNA | Filamin A, alpha |
| IPI00645819.3 | WDR26 | Isoform 2 of WD repeat-containing protein 26 |
| IPI00740142.2 | LOC652147 | U5 small nuclear ribonucleoprotein 200 kDa helicase-like, partial |
| IPI00742682.2 | TPR | Nucleoprotein TPR |
| IPI00783656.1 | MRPL38 | 39S Ribosomal protein L38, mitochondrial |
| IPI00784013.1 | JAK1 | Tyrosine-protein kinase JAK1 |
| IPI00784295.2 | HSP90AA1 | Isoform 1 of Heat shock protein HSP 90-alpha |
| IPI00790292.3 | MRPL45 | cDNA FLJ61100, highly similar to 39S ribosomal protein L45, mitochondrial |
| IPI00794663.1 | TUBA4A | cDNA FLJ58687, highly similar to Tubulin alpha-4 chain |
| IPI00795257.3 | GAPDH | Glyceraldehyde-3-phosphate dehydrogenase |
| IPI00796316.4 | GSN | cDNA FLJ53327, highly similar to Gelsolin |
| IPI00796386.2 | NSRP1 | cDNA FLJ50801, highly similar to Coiled-coil domain-containing protein 55 |
| IPI00807664.2 | MRPL48 | cDNA FLJ60720, highly similar to Homo sapiens mitochondrial ribosomal protein L48 (MRPL48), transcript variant 1, mRNA |
| IPI00854741.2 | LLGL1 | Lethal(2) giant larvae protein homolog 1 |
| IPI00871174.2 | MYCBP | C-Myc-binding protein |
| IPI00893431.2 | EIF3B | cDNA FLJ53410, highly similar to Eukaryotic translation initiation factor 3 subunit 9 |
| IPI00894205.2 | NIPSNAP1 | protein NipSnap homolog 1 isoform 2 |
| IPI00910422.2 | EIF3L | cDNA FLJ52802, highly similar to Eukaryotic translation initiation factor 3subunit 6-interacting protein |
| IPI00922694.1 | HSPA9 | cDNA FLJ51903, highly similar to Stress-70 protein, mitochondrial |
| IPI00926611.1 | IMMT | cDNA FLJ59388, highly similar to Mitochondrial inner membrane protein |
| IPI00930678.1 | SNRPD2 | Small nuclear ribonucleoprotein Sm D2 isoform 2 |
| IPI00937615.2 | EEF1G | Elongation factor 1-gamma |
| IPI00940257.2 | CCT5 | cDNA FLJ52362, highly similar to T-complex protein 1 subunit epsilon |
| IPI00940393.3 | EEF1A1 | EEF1A1 protein |
| IPI00977658.1 | EIF3H | Eukaryotic translation initiation factor 3 subunit H |
| IPI00978796.1 | CFL1 | Cofilin-1 |
| IPI00981317.1 | PPIA | cDNA FLJ75025, highly similar to Homo sapiens peptidylprolyl isomerase A (cyclophilin A) (PPIA), transcript variant 2, mRNA |
| IPI00985384.1 | DDX3X | ATP-dependent RNA helicase DDX3X isoform 3 |
| IPI01008914.1 | EIF4A1 | Eukaryotic initiation factor 4A-I isoform 2 |
| IPI01009129.1 | LLGL2 | cDNA FLJ44733 fis, clone BRACE3026290, highly similar to Lethal(2) giant larvae protein homolog 2 |
| IPI01009811.1 | MRPL1 | 34 kDa protein |
| IPI01010106.1 | KCTD17 | Potassium channel tetramerisation domain containing 17 |
| IPI01011337.1 | PRPSAP2 | cDNA FLJ52841, highly similar to Phosphoribosyl pyrophosphatesynthetase-associated protein 2 |
| IPI01011853.1 | TUBB6 | cDNA FLJ35358 fis, clone PUAEN2000497, highly similar to Tubulin beta-6 chain |
| IPI01013273.1 | CCT6A | cDNA, FLJ79129, highly similar to T-complex protein 1 subunit zeta |
| IPI01015060.1 | MRPL12 | 39S Ribosomal protein L12, mitochondrial |

**Table S2**

| **Gene name** | **Description** |
| --- | --- |
| ADAP1 | arfGAP with dual PH domains 1 |
| AKT1 | v-akt murine thymoma viral oncogene homolog 1 |
| AKT2 | v-akt murine thymoma viral oncogene homolog 2 |
| AKT3 | v-akt murine thymoma viral oncogene homolog 3 (protein kinase B, gamma) |
| AQP9 | aquaporin-9 |
| CBP | cofactor cyclic AMP-binding protein binding protein |
| CCDC115 | coiled-coil domain containing 115 |
| CDC42 | cell division cycle 42 |
| CKAP4 | cytoskeleton-associated protein 4 |
| CREB | cofactor cyclic AMP-binding protein |
| CTGF | [connective tissue growth factor](https://david.ncifcrf.gov/geneReportFull.jsp?rowids=791582) |
| CXCR4 | chemokine (C-X-C motif) receptor 4 |
| DENND5A | DENN/MADD domain containing 5A |
| DYNLL1 | dynein, light chain, LC8-type 1 |
| EGF | epidermal growth factor |
| ERK | extracellular responsive kinase |
| FEZ1 | fasciculation and elongation protein zeta 1 (zygin I) |
| FEZ2 | fasciculation and elongation protein zeta 2 (zygin II) |
| HIST1H1A | histone cluster 1, H1a |
| HIST1H1B | histone cluster 1, H1b |
| HIST3H3 | histone cluster 3, H3 |
| IKBKB | inhibitor of nuclear factor kappa-B kinase subunit beta |
| IL10 | interleukin 10 |
| IL6 | interleukin 6 |
| MAP2K5 | mitogen-activated protein kinase kinase 5 |
| MAPK1 | mitogen-and stress-activated protein kinase 1 |
| MAPK7 | mitogen-activated protein kinase 7 |
| MARCKS | myristoylated alanine-rich protein kinase C substrate |
| MBP | myelin basic protein |
| MCP1 | monocyte chemoattractant protein-1, |
| MMP9 | matrix metallopeptidase 9 |
| MSH2 | mutS homolog 2, colon cancer, nonpolyposis type 1 (E. coli) |
| MSH6 | mutS homolog 6 (E. coli) |
| MYC | v-myc avian myelocytomatosis viral oncogene homolog |
| MYD88 | myeloid differentiation primary response gene 88 |
| NCF1 | neutrophil cytosolic factor 1; neutrophil cytosolic factor 1C pseudogene |
| NCL | nucleolin |
| NCOA3 | nuclear receptor coactivator 3 |
| NFATC2 | nuclear factor of activated T-cells, cytoplasmic, calcineurin-dependent 2 |
| NMT2 | N-myristoyltransferase 2 |
| NUMB | numb homolog (Drosophila) |
| NUP62 | nuclear pore glycoprotein p62 |
| PARD6A | par-6 partitioning defective 6 homolog alpha (C. elegans) |
| PARD6G | par-6 partitioning defective 6 homolog gamma (C. elegans) |
| PAWR | prostate androgen responsive-4 |
| PDPK1 | 3-phosphoinositide dependent protein kinase-1 |
| PIAS4 | protein inhibitor of activated STAT, 4 |
| PPP1R14A | protein phosphatase 1, regulatory (inhibitor) subunit 14A |
| PRKCB | protein kinase C beta type |
| RAF1 | v-raf-1 murine leukemia viral oncogene homolog 1 |
| RELA | v-rel reticuloendotheliosis viral oncogene homolog A (avian) |
| RICTOR | rictor |
| RPS6KB1 | ribosomal protein S6 kinase, 70kDa, polypeptide 1 |
| SP1 | sp1 transcription factor |
| SRC | v-src sarcoma (Schmidt-Ruppin A-2) viral oncogene homolog (avian) |
| STAT6 | signal transducer and activator of transcription 6 |
| TFF1 | trefoil factor 1 |
| TIAM1 | T-cell lymphoma invasion and metastasis 1 |
| TNFRSF1 | tumor necrosis factor receptor superfamily member 1 |
| UTP14A | UTP14, U3 small nucleolar ribonucleoprotein, homolog A (yeast) |
| VHL | von Hippel-Lindau tumor suppressor |
| WWC1 | WW and C2 domain containing 1 |
| XIAP | X-linked mammalian inhibitor of apoptosis protein |
| YWHAB | tyrosine 3-monooxygenase/tryptophan 5-monooxygenase activation protein, beta polypeptide |
| YWHAG | tyrosine 3-monooxygenase/tryptophan 5-monooxygenase activation protein, gamma polypeptide |
| YWHAH | tyrosine 3-monooxygenase/tryptophan 5-monooxygenase activation protein, eta polypeptide |
| YWHAQ | tyrosine 3-monooxygenase/tryptophan 5-monooxygenase activation protein, theta polypeptide |
| ZNF71 | zinc finger protein 71 |
| BCL10 | B-cell lymphoma/leukemia 10 |
| JAM1 | junctional adhesion molecule A |
| MAPK10 | mitogen-activated protein kinase 10 |

**Table S3**

| **Panther Pathway** | **Counts** | **P-Value** | **Genes** |
| --- | --- | --- | --- |
| EGF receptor signaling pathway | 19 | 1.18E-10 | PRKCZ, YWHAZ, YWHAB, PRKCI, RAF1, MAPK10, PRKCB, AKT1, STAT6, CDC42, MAPK1, YWHAG, YWHAH, PPP2CA, YWHAQ, MRPL38, MAPK7, AKT3, AKT2 |
| FGF signaling pathway | 17 | 4.42E-09 | PRKCZ, YWHAZ, YWHAB, PRKCI, RAF1, MAPK10, PRKCB, AKT1, MAPK1, YWHAG, YWHAH, PPP2CA, YWHAQ, MRPL38, MAPK7, AKT3, AKT2 |
| PDGF signaling pathway | 17 | 3.45E-07 | PRKCZ, PRKCI, RAF1, RPS6KB1, MAPK10, PRKCB, AKT1, STAT6, MAPK1, PDPK1, TIAM1, JAK1, MYCBP, MAPK7, MYC, AKT3, AKT2 |
| Apoptosis signaling pathway | 14 | 1.22E-06 | PRKCZ, XIAP, RELA, PRKCI, MAPK10, PRKCB, AKT1, MAPK1, MAPK7, HSPA5, IKBKB, AKT3, HSPA9, AKT2 |
| Parkinson disease | 12 | 4.69E-06 | MAPK1, YWHAZ, YWHAG, YWHAH, YWHAB, YWHAQ, CSNK2B, MAPK10, MAPK7, HSPA5, SRC, HSPA9 |
| T cell activation | 12 | 2.29E-05 | AKT1, CDC42, MAPK1, PRKCZ, PRKCI, RAF1, MAPK10, IKBKB, NFATC2, AKT3, PRKCB, AKT2 |
| PI3 kinase pathway | 11 | 8.96E-05 | AKT1, PDPK1, YWHAZ, YWHAG, YWHAH, YWHAB, YWHAQ, JAK1, RPS6KB1, AKT3, AKT2 |
| Inflammation mediated by chemokine and cytokine signaling pathway | 17 | 0.00028 | PRKCZ, RELA, PRKCI, RAF1, PRKCB, AKT1, STAT6, CDC42, MAPK1, PDPK1, CXCR4, JAK1, MAPK7, NFATC2, IKBKB, AKT3, AKT2 |
| B cell activation | 9 | 0.00028 | MAPK1, PRKCZ, PRKCI, RAF1, IGHV4-31, MAPK10, IKBKB, NFATC2, PRKCB |
| Ras Pathway | 9 | 0.00042 | AKT1, CDC42, MAPK1, PDPK1, TIAM1, RAF1, MAPK10, AKT3, AKT2 |
| Endothelin signaling pathway | 9 | 0.00057 | AKT1, MAPK1, PRKCZ, PRKCI, RAF1, MAPK7, AKT3, PRKCB, AKT2 |
| p53 pathway | 10 | 0.00064 | AKT1, PDPK1, YWHAZ, YWHAG, YWHAH, PPP2CA, YWHAB, YWHAQ, AKT3, AKT2 |
| VEGF signaling pathway | 8 | 0.00094 | AKT1, MAPK1, PRKCZ, PRKCI, RAF1, AKT3, PRKCB, AKT2 |
| Interleukin signaling pathway | 13 | 0.0010 | STAT6, AKT1, MAPK1, IL6, PDPK1, RAF1, MAPK7, IKBKB, MYC, IL10, SRC, AKT3, AKT2 |
| p53 pathway by glucose deprivation | 5 | 0.0023 | AKT1, PPP2CA, RPS6KB1, AKT3, AKT2 |
| Interferon-gamma signaling pathway | 5 | 0.0042 | MAPK1, PIAS4, JAK1, MAPK10, MAPK7 |
| FAS signaling pathway | 5 | 0.0059 | AKT1, GSN, MAPK10, AKT3, AKT2 |
| p53 pathway feedback loops 2 | 6 | 0.0083 | AKT1, PDPK1, PPP2CA, MYC, AKT3, AKT2 |
| Toll receptor signaling pathway | 6 | 0.0095 | MAPK1, MYD88, RELA, MAPK10, IKBKB, TAB1 |

**

**

**Figure supplemental S1.** (A) Survival analysis: The over-expression of PKCζ is associated with poor prognosis. Data were obtained from TCGA database (n= 1445, effect size=0.6523, *P*=0.0011). (B) PKCζ were over-expressed in breast cancer by TCGA database, TPM: Transcripts Per Kilobase of exon model per Million mapped reads (sample size n=1445,Tumor=1118, Normal =327, *P*=0.035).
